# Novel Insights into the Physiology of Nutrient Sensing and Gut-Brain Communication in Surgical and Experimental Obesity Therapy

**DOI:** 10.1007/s11695-023-06739-4

**Published:** 2023-07-20

**Authors:** Lukas D. Frick, Mohammed K. Hankir, Tito Borner, Ermanno Malagola, Bálint File, Daniel Gero

**Affiliations:** 1 Institute of Neuropathology, University Hospital Zurich, University of Zurich, Zurich, Switzerland; 2 Department of Experimental Surgery, University Hospital Würzburg, Würzburg, Germany; 3 Department of Biobehavioral Health Sciences, School of Nursing, University of Pennsylvania, Philadelphia, PA 19104 USA; 4 Division of Digestive and Liver Diseases, Department of Medicine and Irving Cancer Research Center, Columbia University Medical Center, New York, NY 10032 USA; 5 Faculty of Information Technology and Bionics, Pázmány Péter Catholic University, Budapest, Hungary, Institute of Cognitive Neuroscience and Psychology, Research Centre for Natural Sciences, Budapest, Hungary; 6 Wigner Research Centre for Physics, Budapest, Hungary; 7 Department of Surgery and Transplantation, University Hospital Zurich, University of Zurich, Rämistrasse 100, 8091 Zürich, Switzerland

**Keywords:** Obesity, Bariatric surgery, Roux-en-Y gastric bypass, Sleeve gastrectomy, Physiology, Gut-brain communication, Vagal nerve, Tuft cell, Bile acid, Type 2 diabetes mellitus, Bionic technology, Hybrid tissue, Neurostimulation, Brain stimulation, Intestinal millieu, Ingestive behavior, Microbiota

## Abstract

Despite standardized surgical technique and peri-operative care, metabolic outcomes of bariatric surgery are not uniform. Adaptive changes in brain function may play a crucial role in achieving optimal postbariatric weight loss. This review follows the anatomic-physiologic structure of the postbariatric nutrient-gut-brain communication chain through its key stations and provides a concise summary of recent findings in bariatric physiology, with a special focus on the composition of the intestinal milieu, intestinal nutrient sensing, vagal nerve-mediated gastrointestinal satiation signals, circulating hormones and nutrients, as well as descending neural signals from the forebrain. The results of interventional studies using brain or vagal nerve stimulation to induce weight loss are also summarized. Ultimately, suggestions are made for future diagnostic and therapeutic research for the treatment of obesity.

## Introduction

While alterations in intestinal physiology, gut-brain communication, and cerebral connectivity following bariatric surgery (BS) are increasingly recognized as important factors mediating long-term metabolic outcomes [1, 2], the clinical relevance and applicability of recent findings require further investigation. Despite standardized surgical technique and peri-operative care, patients do not evolve uniformly after BS. Excess weight loss (EWL) outcomes after Roux-en-Y gastric bypass (RYGB) or sleeve gastrectomy (SG) vary widely (37.6–94.4%), and existing models of EWL prediction based on demographic data have been shown to be inaccurate [3].

The extent of postbariatric EWL may highly depend on factors that are not regularly assessed during peri-operative clinical care, such as changes in brain connectivity, as documented in studies using functional magnetic resonance imaging (fMRI) [4, 5]. The gastrointestinal (GI) tract senses the chemical, nutritive and volumetric properties of ingested food via a complex gut-brain communication system [6]. As a crucial link between the gut and the brain, the vagus nerve remains an important subject of research in gastrointestinal physiology [1].

This review provides a concise summary of recent findings in bariatric physiology, with a focus on how gut-brain communication pathways are altered following BS and in the context of selected non-surgical experimental weight-loss therapies. The review of specific changes in nutrient sensing in the context of various obesity therapy modalities has the potential to elucidate causes of variability in postbariatric outcomes. The discussion follows an anatomic-physiologic structure along the key stations of nutrient-gut-brain communication to identify potential diagnostic and therapeutic targets for optimizing obesity therapy in the setting of BS. We also explore how integrating biotechnology into BS could amplify the functional brain changes that appear to be crucial for successful weight loss.

## Methods

We performed a narrative review to provide in-depth coverage of nutrient sensing and gut-brain communication in surgical and experimental obesity therapy and to synthesize rationales for future research. Each subchapter has been drafted to provide novel insights into physiologic research from different angles. The selection of included articles from the literature aimed to include the most recent and the most relevant articles from preclinical and clinical research, without the application of a systematic bibliographic search strategy. The articles were critically evaluated based on key results, limitations, suitability of the methods used to test the initial hypothesis, and their impact in the field [7]. Subjectivity in study selection is an inherent limitation of narrative reviews, which may influence the interpretation, the translation and the application of published research [8]. Physiologic changes in nutrient sensing and metabolism following BS are emphasized with italics and summarized in Table 1.

**Table 1:** Physiologic changes in nutrient sensing and metabolism after bariatric surgery discussed in this review

Anatomic station	Physiological parameter	Direction of postbariatric changes
Intraluminal milieu of the small bowel	Postprandial caloric rate and density	↑ [10]
	Gastro-jejunal transit time	↑ [11]
	Bile acid concentration	↑ [149]
	Fat digestion/absorption	↓ [150]
	Intestinal epithelial barrier	↑ stability (↓ absorption of bacterial lipopolysaccharide toxin) [151, 152]
	Glucose transport from the vasculature into the intestinal cells	↑ [153]
	Mitochondrial glucose metabolism	↑ [154]
	Microbiota	↓ obligatory anaerobic Gram-positive bacterial groups; ↑relative abundance of Proteobacteria [33]
Nutrient sensing in the intestinal wall	Number of enteroendocrine cells	↑[9, 20]
	Enteroendocrine cells’ postprandial activity: GLP-1, PYY secretion	↑ [9, 10]
Intestinal branches of the vagus nerve	Transmission of postprandial satiety signals	↑ [1, 55]
Brain control of ingestion	Striate nucleus (reward center)	↑ postprandial dopamine release [118]
	Nucleus of the solitary tract and area postrema (energy status)	↑ stimulation by cytokine growth differentiation factor 15 [90, 93]
	Dorsolateral prefrontal cortex (inhibits ingestion)	↑ stimulation by visual food cues [121]

## Intestinal Nutrient Sensing

Anatomical changes after BS (i.e., RYGB or SG) result in faster delivery of partially undigested nutrients to the jejunum. This, in turn, alters the nutrient-sensing mechanisms controlling gut hormone release (e.g., *increased postprandial GLP-1 and PYY release from enteroendocrine cells (EEC)*) [9, 10]. A longitudinal study showed that after RYGB, patients decreased their meal size in part by lowering the average size, but not the total number of within-meal ingestive bursts [11]. This suggests that neural circuits responsible for within-meal intestinal nutrient sensing are crucial for postbariatric changes in ingestive behavior and demonstrates the rapidity of the gut-brain feedback loop.

A multitude of GI cell types participate in nutrient chemo-sensing and produce signaling molecules to relay this information to other organs. Their composition and distribution across the GI tract vary considerably, indicating that specific nutrient sensing occurs in a tissue-dependent fashion. *EEC and tuft cells* constitute the first line of hormonal, neuronal, and immunogenic response to food intake [12–14]. Nutrient chemo-sensing occurs in part through multiple G protein-coupled nutrient receptors (GPCRs) expressed on the apical membrane of EEC [15], whereas tuft cells participate in chemosensing thanks to their expression of specific taste receptors [16]. Moreover, tuft cells have been shown to regulate immune responses [17] and convey information to the enteric nervous system, eliciting direct neuromodulatory activities [18, 19].

Metabolic disorders such as obesity correlate with major changes in the number of these cells and their hormonal release [9, 20]. Following BS, profound alterations in the distribution and activity of EEC cells have been described [9, 20]. For example, in a comparison of lean control individuals to patients with obesity pre- and post-BS, an overall reduction in the *number of duodenal EEC* was observed in patients with obesity, which was partially restored following SG [9]. Interestingly, individuals with obesity had more mucin-producing Goblet cells, underscoring the biased allocation of the intestinal secretory lineage [21]. Gastrin receptors and the bile acid receptors FXR and TGR5 have been described to participate in this process by modulating EEC differentiation [22–24] as well as intestinal progenitor proliferation [18, 25–27]. Interestingly, these observations suggest that an evaluation of the distribution and activity of secretory cells may provide a valuable readout for proper nutrient chemo-sensing to test the effectiveness of surgical and pharmacological treatment of metabolic disorders.

*Summary:* Multiple cell populations actively participate in postprandial nutrient chemo-sensing. Their localization and function vary across the GI tract, reflecting an anatomical regionalization in nutrient sensing. Metabolic disorders can alter the representation and function of these cells, leading to improper chemo-sensing. BS appears to be effective in counter-balancing these obesity-related alterations.

## Intestinal Milieu

The internal environment of the GI tract is shaped by numerous factors, including diet, digestive secretions, resident microbiota, and fungi [28–33]. Recent developments in ingestible, pH-sensitive sampling devices have revealed profound differences in these factors throughout the GI tract in healthy humans [34, 35], which are all liable to change following the anatomic alterations of BS. This may, in turn, contribute to postbariatric metabolic outcomes, such as EWL, or the weight loss-independent amelioration of liver steatosis and type 2 diabetes mellitus (T2DM).

Rodent models of BS have been instrumental in identifying post-surgical changes in the intestinal milieu, particularly in gut microbiota and bile acids, which are interrelated and play emerging roles in metabolic health [36]. For example, a study on Zucker fatty rats found that RYGB causes major shifts in the microbiota across the small and large intestine [37]. Interestingly, transplanting ileal microbiota from RYGB-operated rats to germ-free mice worsened oral glucose tolerance [37], likely due to the generation of metabolites that disrupt the intestinal epithelial barrier and trigger *systemic endotoxemia* [38]. On the other hand, transplanting colonic microbiota of RYGB-operated rats to germ-free mice improved oral glucose tolerance, possibly by stabilizing the intestinal epithelial barrier through increased bile acid receptor FXR signaling via the generation of secondary bile acids [39]. These findings suggest that the changes in gut microbiota and their associated metabolites after RYGB may have opposing effects on glycemic control, depending on the gut region [40]. For SG, cecal and fecal levels of the *primary bile acid cholic acid-7 sulfate (CA7S)* have been found to increase in mice and patients after surgery [41]. Moreover, oral administration of CA7S improved glucose tolerance in mice in a TGR5-dependent manner [41]. These findings illustrate how understanding changes in the gut milieu after BS can guide the development of new pharmacological treatments for T2DM. There is also evidence that the *gut microbiota* plays a role in the reduced food intake [41] and protection from weight gain after RYGB in diet-induced obese rats [42]. In line with this, antibiotic-based depletion of the gut microbiota abrogates the effects of RYGB on energy balance, and fecal transplantation is effective in transferring the metabolic phenotype of RYGB-operated rats to diet-induced obese rats, again mediated by increased FXR signaling [42]. These findings appear to have limited translational value, since transferring human feces after RYGB fails to impact body weight in rodents and in humans [43–45]. Interestingly, this intervention does improve oral glucose tolerance in recipients through reduced glucose absorption and increased adipose tissue glucose utilization [43, 44], suggesting that post-RYGB changes in gut microbiota mainly improve glycemic control. Clearly, the impact of RYGB on gut microbiota-host interactions is complex, and more research is needed before an efficient RYGB microbiota-based treatment can be developed for patients with metabolic syndrome.

Beyond bile acids and microbiota, BS affects other factors in the intestinal milieu. In a study of women with T2DM and obesity, samples were collected by endoscopy from the stomach and throughout the small intestine two weeks before and three months after RYGB, providing insight into how glucose metabolites change in the intestinal milieu [46]. As revealed by mass spectrometry analysis, RYGB led to reduced levels of aromatic and branched-chain amino acids, while it increased the metabolism of phenylacetate and degradation of trehalose in the duodenum and jejunum, and degradation of lactose in the ileum [46].

*Summary:* Preclinical and clinical research is beginning to shed light on the complex changes in the intestinal milieu after BS [47]. Further work is needed to determine how this influences circulating signaling molecules that communicate with peripheral tissues and the brain to improve metabolic health.

## Nutrient Sensing Via the Vagus Nerve

The vagus nerve provides parasympathetic innervation of the gastrointestinal tract from the esophagus to the splenic flexure of the colon. It carries efferent signals from the dorsal vagal nucleus in the hindbrain, which are integrated by the enteric nervous system to control smooth muscle contraction and glandular secretion. However, afferent vagal fibers vastly outnumber efferent fibers [48]. Vagal afferent neurons, whose cell bodies reside in the inferior (nodose) ganglion of the vagus nerve in the jugular foramen, carry sensory information from the gut to the nucleus of the solitary tract (NTS) of the hindbrain.

In the stomach, most vagal afferents terminate in intramuscular arrays within the circular and longitudinal muscle layers, as well as in the ganglia of the myenteric plexus. They are thus well-positioned to react to *mechanical stretch and tension* [49]. A smaller number of vagal afferent fibers project to the gastric mucosa and respond to stroking [50]. In the intestine, most vagal fibers are *chemosensitive* and end in the mucosa, where they respond to nutrients and gastrointestinal hormones [51, 52]. Recently, new genetic tools – including monosynaptic neural tracing, optogenetics, and chemogenetics – allowed the characterization of vagal afferents in mice that rapidly communicate the presence of nutrients from the gut to the brain. In one study, a novel neuroendocrine cell that communicates the presence of sugars to vagal afferents via glutamate was identified. Because of the synaptic-like nature of this signaling, the authors termed this neuroepithelial unit a “*neuropod”* [53].

Signaling through the vagal nerve may mediate some of the benefits of BS [54, 55]. In RYGB, only the dorsal and ventral gastric vagal branches supplying the stomach are severed while forming the gastric pouch. However, the vagal branches traveling with the gastroduodenal and superior mesenteric arteries remain intact. Thus, vagal innervation of the intestine, liver, and pancreas can be mostly spared, except for some branches traveling along the lesser curvature of the stomach to reach the proximal duodenum and parts of the pancreas [56]. Evidence from the rat model indicates that preservation of the celiac branches of the vagal nerve enhances weight loss after RYGB [57–59].

While gastrointestinal hormones can reach the brain via the circulation to directly regulate feeding behavior, afferent vagal nerve endings contain receptors for many intestinal hormones, and signaling via the vagus nerve may partly account for their effects [60]. Cholecystokinin (CCK), which is released by enteroendocrine cells after ingestion of protein or fat, binds to CCK-A receptors on vagal afferents to suppress further food intake [61]. In rodent models, however, a complete transection of the abdominal vagus nerve has a negligible effect on body weight, although it leads to an increase in meal size, which is compensated by reduced meal frequency [62]. Thus, *humoral regulation may be sufficient for long-term control of body weight*, with the *vagus nerve being more important in the short-term regulation of feeding*. However, if a complete transection of all vagal afferents and efferents is performed, it is impossible to identify the contributions of individual neuronal subpopulations, which may have different and opposing effects [63]. In obesity, the sensitivity of vagal afferents to CCK signaling, as well as to mechanical distension of the gut, is reduced [64, 65]. Therefore, targeting specific vagal populations to heighten the response to satiety signals remains a promising therapeutic approach.

*Summary:* The vagus nerve is instrumental in rapidly responding to satiety signals such as mechanical stretch, intraluminal nutrients and caloric concentration, as well as to paracrine gastrointestinal hormones. However, its role in long-term weight control remains less defined, necessitating a more detailed exploration of vagal signaling and plasticity in obesity and in post-BS conditions.

## The role of the Hindbrain in the Hypophagic Effects of Bariatric Surgery

Historically, the mediobasal hypothalamus has been considered the main sensor of the metabolic state and the integrator of effector actions aiming at an optimal body weight [66]. However, the hindbrain and the limbic system are other key brain areas implicated in body weight-regulating mechanisms that serve as alternative targets for weight loss therapies [66]. The NTS and the area postrema (AP) are two adjacent and highly interconnected hindbrain structures that process information on peripheral energy status from circulating signals and branches of the vagus nerve [67]. The presence of fenestrated capillaries and the lack of tight junctions between endothelial cells allow neurons in the AP/NTS to be reached by hormonal and nutrient signals that cannot cross the blood–brain barrier in other brain regions [68, 69]. AP/NTS neurons express a variety of receptors and directly respond to nutrients (e.g., glucose) and several peptides produced peripherally that play a direct role in the regulation of feeding and homeostasis, e.g., GLP-1, amylin, leptin, and ghrelin [70–73].

The AP/NTS is also a key hub for the integration of pathological modulators of energy balance. Increased neuronal activity in this area is associated not only with physiological satiation, but also with emesis and nausea [74, 75]. AP/NTS neurons project to a number of regions involved in the regulation of feeding [76–80]. Among these connections, many studies highlight the importance of NTS lateral parabrachial nucleus (lPBN) projections [67, 81–84], where calcitonin gene-related peptide (CGRP) mediation of anorexia and malaise occurs.

Postprandial nausea and vomiting represent potential complications of BS [85]. Although recent clinical neuroimaging and animal studies have explored the effects of BS on forebrain function [86], relatively few studies have focused on the role of the caudal hindbrain and its contribution to BS outcomes. It has been shown in preclinical models that vagal afferent signaling is required for optimal weight loss and diminished fat preference following RYGB [58]. Relatedly, it was demonstrated that in RYGB mice, eating a voluntary meal induced exaggerated expression of the marker of neuronal activation c-Fos in the AP/NTS [87]. Interestingly, a significant portion of the activated neurons in the lPBN expressed CGRP, suggesting the involvement of the hindbrain in the mediation of the food taste-visceral malaise association occurring after surgery [87]. SG also increases nutrient-induced c-Fos expression in the AP/NTS compared to control animals [88]. These results may provide a mechanistic explanation for such post-surgical symptoms as nausea, vomiting, and visceral malaise, which most frequently occur after the consumption of large meals rich in fat or sugar [89].

The gut-generated signals responsible for the modified hindbrain response after RYGB are largely unknown. However, recent research has focussed on the stress-response cytokine *growth differentiation factor 15 (GDF15)* and its body weight-suppressive effects. GDF15 is an inflammatory biomarker released by various tissues [90–94], and elevated circulating levels of GDF15 are associated with anorexia, malaise, and cachexia in a variety of diseases and physiological states [95–105]. GDF15 acts as a ligand on a highly localized hindbrain G-family α-like receptor [103, 106–109]. Exogenous administration of GDF15 induces nausea and emesis, suggesting malaise and conditioned food aversion as key components of GDF15-induced anorexia [110–113].

Interestingly, while circulating GDF15 levels increase only slightly in obesity, they are substantially elevated in patients following RYGB (and, to a lesser extent, SG) [90–94]. Importantly, there was a clear correlation between GDF15 levels and the magnitude of post-BS EWL [90, 93]. In mice treated with GDF15, there was a reduced preference for a high-fat diet [114]. Notably, RYGB-operated rats have increased circulating and portal vein GDF15 levels, and this is negatively correlated with their food intake and body weight [40]. While a separate study also showed increased circulating GDF15 levels and GDF15 protein in the gastric pouch, jejunum, and ileum of RYGB-operated rats [115], more data is needed to establish a causal link between gut-derived GDF15 and the beneficial effects of BS [116, 117].

*Summary:* Despite being a relatively new and rapidly evolving field, emerging evidence strongly suggests that GDF15 plays a crucial role in inducing hypophagia in various medical conditions by primarily triggering feelings of malaise through signaling to the AP/NTS. However, further pre-clinical and clinical research is needed to fully understand its role in the positive outcomes (e.g., weight loss) and negative effects (i.e., GI malaise) of bariatric surgery.

## Weight Loss by Neurostimulation

Based on the strong relationship between BS and brain activity, several studies tried to induce weight loss by direct manipulation of the brain [118]. Cortical functions implicated in the development of obesity include reward, attention, emotional regulation, impulsivity, and motivation. People with obesity show decreased inhibitory control (also known as response inhibition to environmental stimuli) and impaired memory systems [119]. Postbariatric weight loss correlates with functional changes in brain regions associated with cognitive functions altered by obesity [120]. Food cue-based neuroimaging studies after BS have shown increased activation in the *dorsolateral prefrontal cortex (DLPFC)*, which is responsible for inhibitory control, while brain regions responsible for memory and reward processes, such as the hippocampus and insula, are less active [121–123]. In an attempt to stimulate weight loss, neuromodulation techniques, such as deep brain stimulation (DBS), transcranial magnetic stimulation (TMS), transcranial direct current stimulation (tDCS) of cortical and subcortical locations, neurofeedback and peripheral nerve stimulation/blockade have been applied. 

*Invasive DBS* treatment for weight loss has been limited to only a few patients. These studies aimed to stimulate the hypothalamus or the nucleus accumbens and achieved only clinically irrelevant EWL in humans [124–126]. It should be noted that the microlesions in the brain during DBS electrode placement can also evoke metabolic changes [127], and thus, it is not clear whether the altered functionality is the consequence of the stimulation. In addition, the lack of a standardized protocol for the DBS studies makes it hard to evaluate their efficacy [128]. Very recently, a DBS clinical pilot was conducted on two patients, where the electrophysiological signatures of food craving were used to activate implanted DBS to enhance inhibitory control [129]. After six months, the BMI of both subjects decreased by 2–3 kg/m^2^, which is clearly inferior to what can be achieved with BS [130].

*High-frequency repeated TMS* is a method used for selective and non-invasive excitatory stimulation of the cortex. A few studies have shown that TMS stimulation of the DLPFC is able to induce weight loss [131–133]. Although the results were statistically significant, the achieved weight loss (BMI change < 1 kg/m^2^ / weight-loss 2–3 kg) is massively smaller than the one observed post-BS. *Non-invasive tDCS* paradigms aim to interfere with cortical systems at the DLPFC to increase the inhibitory control of ingestion. Based on a meta-analysis, a consistent decrease in food craving and energy intake could be achieved [134, 135]; however, no EWL was seen [136, 137]. Besides external stimulation paradigms, patients can learn to effectively manipulate the activity of circumscribed brain areas with *online neurofeedback*. These treatments are based on the voluntary regulation of brain activity feedback via real-time EEG [138]. Studies using EEG [139] and fMRI paradigms [140] introduced the possibility of volitional regulation of frontal brain activity. However, research showing a relationship between successful neurofeedback and weight loss is still lacking.

Directly targeting the vagal nerve appears to be more effective for weight loss than cortical manipulation of the gut-brain axis. *Vagal nerve stimulation (VNS)* involves the surgical implantation of electrodes in the neck and a generator under the skin below the clavicle to provide electrical stimulation to the vagus nerve. The treatment has achieved reduced food intake and EWL proportional to the initial BMI [141, 142]. The opposite approach – *electrical vagal nerve blockade* – is also established as a potential treatment for obesity. The ReCharge trial investigated a vagal blocking device, which employs electrodes placed on the anterior and posterior vagal trunks close to the oesophageal junction, through which an alternating current was applied to block vagal nerve signaling for 12 h per day. One-year EWL was significantly greater with vagal blocking compared to sham treatment (24.4% vs. 15.9%) [143], and this effect remained quite stable at 2-year follow-up [144], with associated metabolic improvements.

*Stimulation of intestinal sensory cells* and vagal afferents by electrodes, hybrid or “bionic” tissues may offer a new therapeutic avenue for regulating satiety [145] and treating obesity. The mechanism of action of intestinal stimulation is not completely understood, but is likely manifold, including a) an accelerated intestinal transit which reduces fat absorption and enhances the nutrient-induced release of GLP-1 and GIP in the distal ileum, and b) an increased expression of hypothalamic oxytocin-immunoreactive positive neurons, which may have a direct effect on adipocytes or an indirect effect in promoting lipolysis [146, 147]. Preclinical data are promising: intestinal electrical stimulation has been shown to reduce food intake in rats and pigs and to reduce intestinal absorption and body weight, as well as to improve glucose tolerance and insulin sensitivity in rats [146, 148]. The first human feasibility study included 9 participants, used laparoscopically implanted duodenal stimulation electrodes, and showed effectiveness in optimizing glycemic control and high-density lipoprotein levels [148].

*Summary:* Neurostimulation of cortical and subcortical areas may lead to weight loss, but the effect size remains relevantly below the observed weight loss achieved after BS. However, stimulation of the peripheral nervous system and hormone-producing cells of the small bowel or intermittent blockade of the abdominal branches of the vagal nerve may open potential pathways to be explored in the quest for non-surgical or adjuvant metabolic therapies.

## Future Outlook

The gut-brain hormonal and synaptic communications, together with GI microbiota, are among the key components for the treatment of obesity. The beneficial postbariatric shifts in the intestinal microbiota and their metabolites, of cellular changes in the GI mucosa, of hormonal, cytokine and vagal signal transmission have been demonstrated.

Future studies should focus on investigating how these different pathways interact and on the neurohormonal changes driven by the accelerated delivery of less digested food into the small bowel following BS. Longitudinal studies can track changes in brain activity and connectivity over time, helping to identify causal relationships between brain activity and postbariatric weight loss. Directed network models can identify key hubs in these systems that might be targeted for therapeutic interventions. Additionally, recent advances in tissue clearing and light-sheet microscopy may enable the study of the GI nervous system (e.g., the vagus nerve and enteric nervous system) *in toto*, allowing for further elucidation of the role of vagal afferents in regulating food intake.

Together with drug-based treatments of obesity, the development of biomedical electronic implants, reaching the micro- and nanoscale, may also provide a therapeutic avenue for the treatment of metabolic disorders [145]. The development of obesity treatments that mimic BS, but are less invasive and are more easily scalable to the eligible patient population, is a high priority. Currently, bionic intestinal stimulators are not available for human use and their potential clinical efficacy is merely hypothetical, and the transferability of preclinical observations to human practice remains to be proven. Limitations and challenges of non-surgical metabolic therapies stem from the chronic nature of obesity and related diseases. Non-surgical weight-loss treatments would ideally need to replicate the pleiotropic physiologic changes and the meal-triggered neurohormonal responses observed after BS. These goals are mainly constrained by the cost of lifelong conservative therapy, by patient compliance, and by the long-term efficacy and side-effect profile of any new treatment. Another limitation is related to the genetic and social factors that may increase the predisposition to obesity, which have not been addressed in this review.

The modified GI anatomy after BS provides a perfect model to study changes at each station of the ingestive signal transmission chain, from the intestinal milieu to the brain. There is a need for further novel and real-time diagnostic studies to better unravel the physiologic mechanisms of BS, with the ultimate goal of broadening preventive and therapeutic strategies for obesity.
